# Postoperative Physiotherapy Approach for a Rare Case of Adult Ileocecal Intussusception

**DOI:** 10.7759/cureus.29668

**Published:** 2022-09-27

**Authors:** Nikita H Seth, Chaitanya A Kulkarni, Shubhangi Patil, Om C Wadhokar

**Affiliations:** 1 Physiotherapy, Ravi Nair Physiotherapy College, Wardha, IND; 2 Community Health Physiotherapy, Datta Meghe Institute of Medical Sciences, Wardha, IND; 3 Community Health Physiotherapy, Ravi Nair Physiotherapy College, Wardha, IND; 4 Musculoskeletal, Ravi Nair Physiotherapy College, Wardha, IND

**Keywords:** intussusception, laparoscopy, physiotherapy, hemicolectomy, case report

## Abstract

We report a rare case of a 32-year-old male diagnosed with a mass of intussusception in the right lumbar region. Adult intussusception has a prevalence of less than 5%. Among all cases of intestinal obstructions, adult intussusception is found to be only 1-3%. Adult intussusception of the bowel is uncommon. In contrast to intussusception in children, the traditional trio of palpable sausage, jelly stools, and discomfort is rarely seen. Adults usually present with nonspecific findings that last for a long time. We share the case of a patient who presented with complaints of pain in the abdomen and loss of appetite for six months. In addition, the patient also complained of constipation. The patient had a history of easy fatigue on moderate-intensity activity. Ultrasonography of the abdomen was suggested which revealed bowel wall thickening in the subhepatic region, with likely intussusception and formation of a lump with few enlarged lymph nodes adjacent to it. A confirmatory diagnosis was made after a computed tomography scan. In all cases, surgical intervention is required, and an organic lesion inside the invaginated section of the colon is discovered to be the lead point in up to 90% of cases. The laparoscopic procedure can be used for both diagnostic and therapeutic purposes. A right hemicolectomy was performed through a vertical midline incision. The patient was referred to the physiotherapy department for further management.

## Introduction

A spontaneous invagination of a section of the intestine into another intestinal loop is referred to as intussusception. Although it is more common in youngsters, it is responsible for 1-5% of intestinal blockages in adults. Adult intussusception can be difficult to diagnose due to the wide range of symptoms [1]. It can be a chronic, intermittent, or acute disorder. Adults rarely develop intussusception of the intestine. In contrast to intussusception in children, the traditional trinity of palpable sausage, jelly stools, and discomfort is rarely seen in adults. Adults present with a variety of nonspecific symptoms that last a long time. Intussusception of the large bowel in adults was linked to cancer in 70% of cases. Intussusceptions of the small bowel present with ileocecal and colon benign lesions [2].

Intussusception occurs when a lesion in the intestinal wall causes invagination by disrupting normal peristalsis. It can develop in any part of the small or large intestine. Enteroenteric, appendiceal-ileocolic, ileocolic, appendiceal, rectoanal, colocolic, and stomal intussusceptions are named from the locations of the intussusceptum and intussusceptions in the bowel, namely, enteroenteric, appendiceal-ileocolic, ileocolic, colocolic, rectoanal, and stomal intussusception [3].

Because of their lack of mobility, redundancy, and anatomic fixation, the upper gastrointestinal structures, notably the esophagus, stomach, and duodenum, are rarely involved in intussusception. Furthermore, the most prevalent sites are at the intersections of freely moving segments and immovable areas, such as the retroperitoneum or adhesions [4].

In all cases, surgical intervention is required, and an organic lesion inside the invaginated section of the colon is discovered to be the lead point in up to 90% of cases. Radiologic or surgical results are used to make a diagnosis. The results of a computed tomography (CT) scan are pathognomonic and can detect intussusception in about 72% of cases. The laparoscopic procedure can be used for both diagnostic and therapeutic purposes. In adults with intussusceptions, laparoscopy may be utilized as the final diagnostic or therapeutic tool [5].

## Case presentation

Patient information

A 32-year-old male patient presented to our hospital with a six-month history of pain in the abdomen following food ingestion, loss of appetite, and constipation. There was no history of hematemesis, nausea, and vomiting. The patient had an alcohol addiction for 15 years. Physical assessment revealed chronic conjunctival pallor, afebrile, blood pressure of 100/70 mmHg, and heart rate of 98 beats per minute. There was grade 2 abdominal tenderness with no distension, and there was a palpable abdominal lump of approx 4 × 4 cm on the right lumbar region on deep palpation. Laboratory investigations showed macrocytic anemia (hemoglobin, 5.7 g/dL; normal: 12-16 g/dL). Four units of whole blood, normal saline, and ringers lactate were used to resuscitate the patient.

Clinical findings

The patient was well oriented to time, place, and person. On local examination, a distended abdomen with mild-to-severe soreness is discovered during a physical examination (consistent with parietal peritoneal irritation). It is possible that one may have decreased or absent bowel noises and an abdominal lump. If the patient presents late in the disease course, symptoms of peritonitis or intestinal ischemia may be present, as well as discomfort that is out of proportion to physical examination findings. On respiratory assessment, the patient had grade 1 breathlessness on modified Medical Research Council MMRC, which was gradual in onset, with walking being an aggravating factor, and on rest, the patient felt relief. The patient also had a history of early fatigue. There was reduced chest expansion. In addition, the patient was hypotensive. There can be various differential diagnoses, due to nonspecific findings of rare causes of such symptoms that might be seen in adult intussusception. On laboratory investigations, nonspecific inflammatory markers were raised including C-reactive protein and thrombocytosis.

Diagnostic assessment

In adult and pediatric cases, the assessment of intussusception is different. Intussusception in children is usually idiopathic and benign, and a high index of suspicion can help with diagnosis. The ultrasound scan is preferred for diagnosing intussusception in children due to its specificity. The classical feature is the doughnut sign formed centrally and the outer ring formed by edematous intussuscipiens. Massive air in cases of intestinal distension or morbid obesity, which might result in a lower rate of detection and diagnosis of intussusception, is the limitation of using this modality [6].

**Figure 1:**
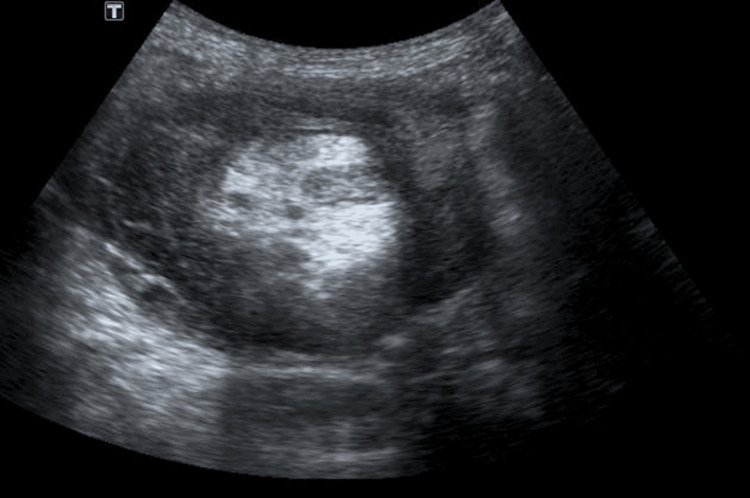
Ultrasonography of the abdomen.

**Figure 2:**
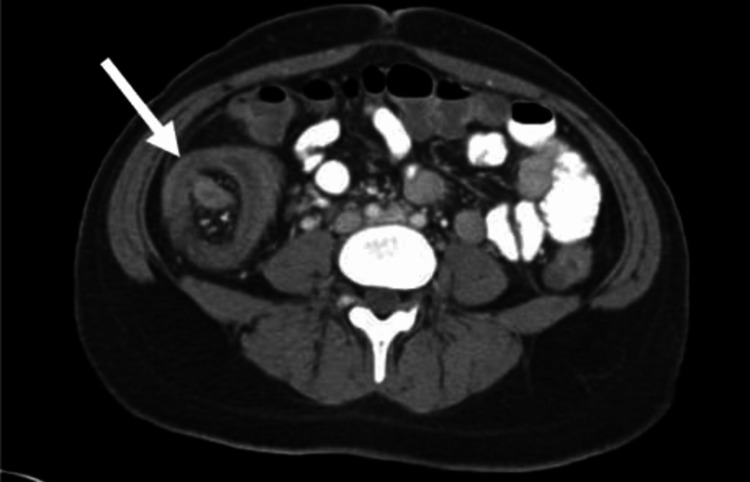
Computed tomography scan imaging.

Ultrasonography of the abdomen and pelvis revealed bowel wall thickening in the subhepatic region with likely intussusception and formation of a lump, as well as a few enlarged lymph nodes adjacent to it with mild hepatomegaly (Figure 1). On CT scan, mass of intussusception in the right lumbar region measured 70 × 66 mm, with pericolic lymph nodes, with the largest measuring 20 × 20 mm. No other significant abnormality was detected (Figure 2).

A timeline of the events is presented in Table 1.

**Table 1:** Timeline of events.

Event	Date of event
Date of admission	20/4/2022
Date of operation	27/4/2022
Date of discharge	9/5/2022

Intervention

**Figure 3:**
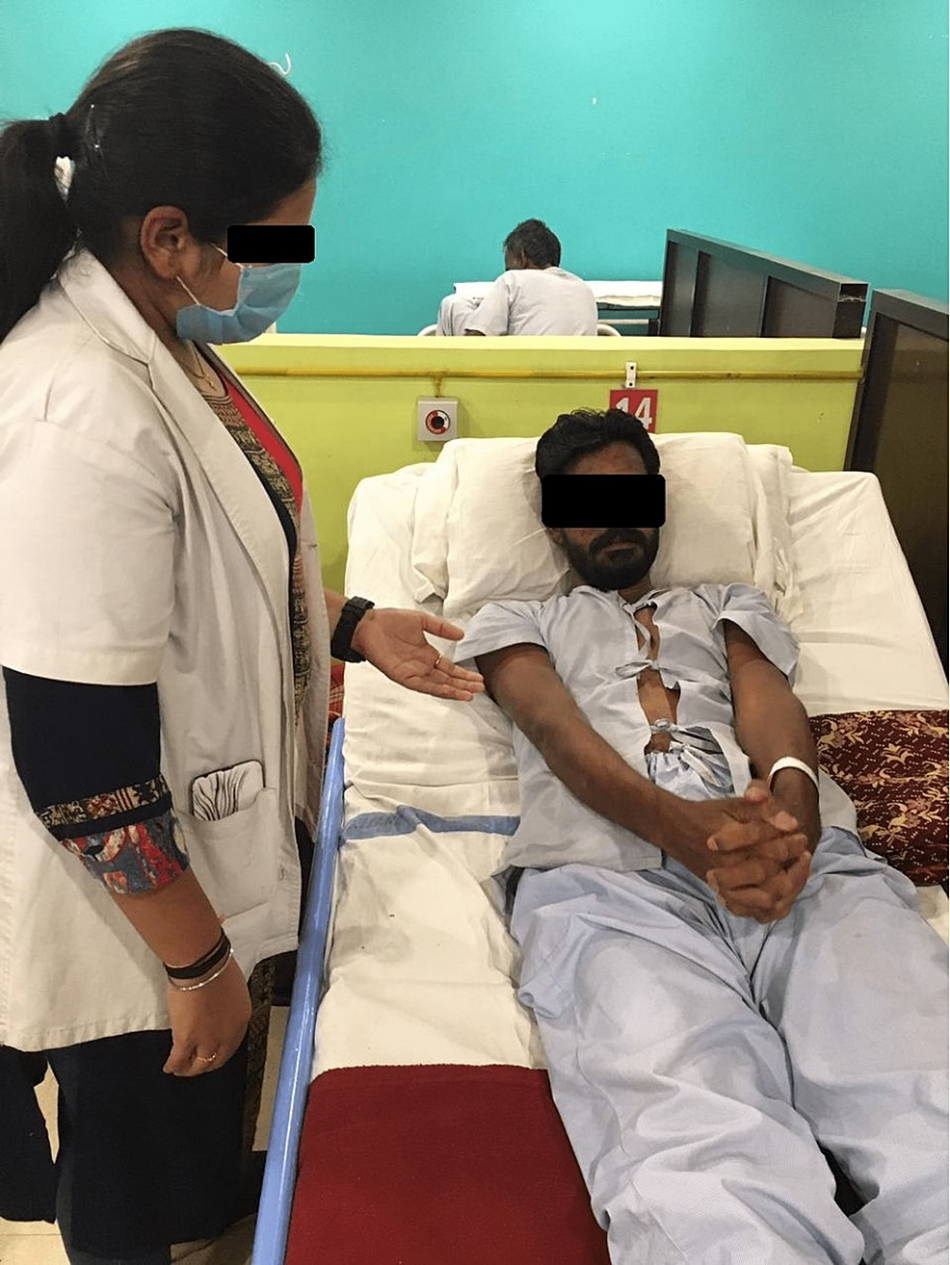
Teaching thoracic expansion with purse lip breathing.

Adult intussusceptions have traditionally been treated surgically due to the presence of a pathology that serves as a lead point. However, more recently, extensive use of CT/magnetic resonance imaging has led to an increase in the radiographic identification of intussusception, which can be associated with ambiguous gastrointestinal symptoms or none at all. The patient underwent a laparoscopy. Both the cecum and ascending colon were hemorrhagic and thickened. The appendix was invisible [7]. To treat the ischemic colon caused by intussusception, a laparoscopic right hemicolectomy was performed. Because the colon had been damaged, the intussusception was not reduced intraoperatively. Cecum pathology revealed hemorrhage, acute inflammation, and ulceration. The lead point was discovered to be an ileocecal valve lipoma. The recovery period was unremarkable. By the second postoperative day, the patient’s bowel function had returned, and he was passing flatus. He was discharged on the 11th postoperative day. Goal-directed physiotherapy rehabilitation is mentioned in Table 2 and Figure 3.

**Table 2:** Physiotherapy rehabilitation.

Goal	Intervention	Rationale
Patient education	Lifestyle modification: quit alcohol	To improve the quality of life and stay healthy
To teach the patient about precautions of the suture site	Splint coughing. Looking for the signs of infection, that is, redness and oozing	Prevents the opening of sutures and surrounding soft-tissue injury
To improve endurance and prevent post-anesthetic complications	Breathing exercises, pulse lip breathing, and thoracic expansion exercise	Maintains airway patency and improves lung capacity
To improve the strength of the abdominal and back muscles	Static abdominals and static backs	Helps to achieve good posture and prevent secondary complications

## Discussion

Adult intussusceptions are uncommon. Nash et al. published a multicenter six-patient case series of adult intussusceptions that found no case of the colocolonic variety, while, in contrast, found four out of 791 documented cases of adult intussusception in adult cystic fibrosis patients, representing a 0.5% prevalence. Ileocolic intussusceptions account for 80-95% of neonatal intussusceptions and can resolve spontaneously, whereas colocolonic intussusceptions are uncommon. As a result, the likelihood of spontaneous resolution cannot be predicted. Intussusception due to severe constipation has been linked to pathological lead points such as inspissated secretions, epithelial damage, increased lymphoid follicles, appendix distension, and distal intestinal obstruction syndrome [7].

In this case, the patient had no typical complaints except abdominal pain along with a history of alcohol abuse for the last 15 years and was diagnosed with rare ileocecal intussusception. The limitation of the case report was not being able to get enough data for correlating the alcohol history with the presenting symptoms [8].

## Conclusions

Intussusception is a common and often benign ailment in children that may usually be treated without surgery. However, the illness is uncommon in adults, and it can be difficult to diagnose due to nonspecific symptoms. Despite their rarity, most general and colon and rectal surgeons treat intussusception patients, and the best results can only be accomplished if the principles stated earlier are understood.
